# Planning the Follow-Up of Patients with Stable Chronic Coronary Artery Disease

**DOI:** 10.3390/diagnostics11101762

**Published:** 2021-09-25

**Authors:** Guillermo Romero-Farina, Santiago Aguadé-Bruix

**Affiliations:** 1 Cardiology Department, Hospital Universitari Vall d’Hebron, Institut de Recerca (VHIR), CIBERCV, Universitat Autònoma de Barcelona, 08193 Bellaterra, Spain; 2 Department of Nuclear Medicine, Hospital Universitari Vall d’Hebron, Institut de Recerca (VHIR), CIBERCV, Universitat Autònoma de Barcelona, 08193 Bellaterra, Spain; saguade@vhebron.net

**Keywords:** chronic coronary artery disease, planning the follow-up, cross-sectional analysis, longitudinal analysis

## Abstract

Cardiovascular disease remains the leading cause of death among Europeans, Americans, and around the world. In addition, the prevalence of coronary artery disease (CAD) is increasing, with the highest number of hospital visits, hospital readmissions for patients with decompensated heart failure, and a high economic cost. It is, therefore, a priority to try to plan the follow-up of patients with stable chronic CAD (scCAD) in relation to the published data, experience, and new technology that we have today. Planning the follow-up of patients with scCAD goes beyond the information provided by clinical management guidelines. It requires understanding the importance of a cross-sectional and longitudinal analysis in the clinical history of scCAD, because it has an impact on the cost of healthcare in relation to mortality, economic factors, and the burden of medical consultations. Using the data provided in this work facilitates and standardizes the clinical follow-up of patients with scCAD, and following the marked line makes the work for the clinical physician much easier, by including most clinical possibilities and actions to consider. The follow-up intervals vary according to the clinical situation of each patient and can be highly variable. In addition, the ability to properly study patients with imaging techniques, to stratify at different levels of risk, helps plan the intervals during follow-up. Given the complexity of coronary artery disease and the diversity of clinical cases, more studies are required in the future focused on improving the planning of follow-up for patients with scCAD. The perspective and future direction are related to the valuable utility of integrated imaging techniques in clinical follow-up.

## 1. Introduction

The number of patients with coronary artery disease (CAD) has increased. An estimated 15.5 million American adults have chronic CAD, and more than 7 million have angina [1,2]. Furthermore, the presence of chronic angina approximately doubles the risk of major cardiovascular events [1,2]. In Europe, cardiovascular disease is the most important cause of death (42%) for women, CAD corresponded to 20%. Cardiovascular disease is also the most important cause of death (51%) for men, CAD corresponded to 21% [3].

**Figure 1:**
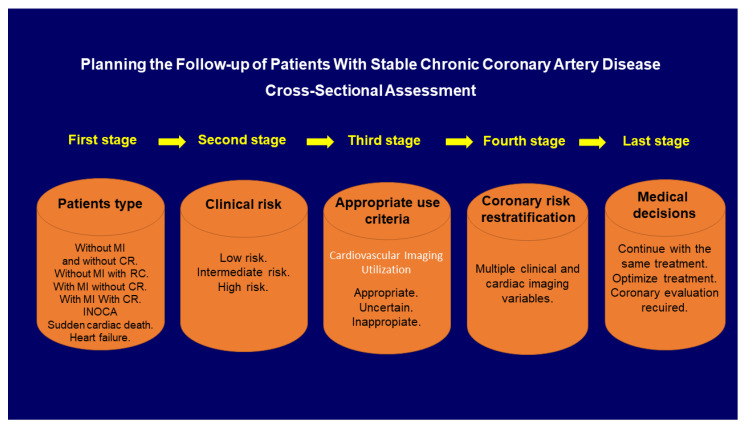
Cross-sectional assessment algorithm to evaluate patients with stable chronic coronary artery disease. Five-stage assessment. Every time that patients have a medical check-up during the follow-up, they require this cross-sectional assessment, although not all patients in the clinical practice setting need to be evaluated for all these steps. For example, if a patient during the follow-up continued to be low risk, there would be no need to continue with stages 3, 4 and 5.

**Figure 2:**
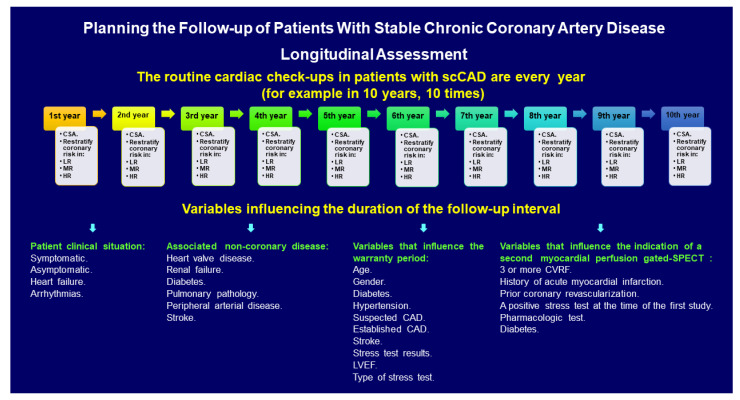
Longitudinal assessment algorithm. If we consider an annual cardiac check-up (Cch-up), as usual, the average number of Cch-ups during 10 years of follow-up is approximately 10. In this figure, many variables that can affect or determine the number of Cch-ups and/or the time intervals between Cch-ups are observed. Thus, the follow-up intervals vary according to the clinical situation of each patient. As clinicians, we know that there are patients who require check-ups every 6 months, or less (patients with MR or HR). Interval could be highly variable. If there is a possibility of adequately studying patients with imaging techniques, in order to be able to stratify them at different risk levels, this will help to plan the intervals during follow-up. CSA, cross-sectional assessment; LR, low risk, MR, moderate risk; HR, high risk.

Planning the follow-up of patients with stable chronic CAD (scCAD) from a clinical and cardiac imaging point of view is a complex issue that is usually poorly developed in the current literature. All the clinical guidelines that currently exist in relation to scCAD refer to the diagnosis, prognosis, appropriate application criteria, and the therapeutic management of scCAD. Currently, there are no clearly defined guidelines for the follow-up planning of patients with scCAD. Although, if not managed properly, poor follow-up can lead to higher mortality, morbidity, and healthcare costs with higher hospital readmission recurrence rates and higher repeated coronary angiogram rates. Planning is influenced by multiple aspects (Figure 1 and Figure 2) that affect cost, cardiology department organization, and the burden of care. For the study of patients with scCAD, we have multi-modality imaging (stress echocardiography, radionuclide myocardial perfusion imaging, cardiac magnetic resonance, coronary computed-tomography angiography, and invasive coronary angiography) that each center will apply in relation to their experience and availability. We tried to show that the assessment and planning of the follow-up of patients with scCAD depends on all the steps presented in Figure 1. In this manuscript, we intend to organize the information and tools that we currently have in order to establish a study methodology in the follow-up of patients with scCAD.

## 2. Stages in Planning Follow-Up

During the follow-up of patients with scCAD, we are constantly evaluating the risk of new cardiac events (re-stratification), the progression of the disease in vessels with atherosclerotic involvement, or the appearance of the disease in vessels not yet affected.

Why is it advisable to plan the follow-up in patients with scCAD?

One of the main reasons is because these patients must be constantly alert to the appearance of new cardiac events and/or progression of CAD from an anatomical and functional point of view. In clinical practice and in most patients, the first examination to detect myocardial ischemia is the exercise test, while from a functional and non-anatomical point of view, nuclear cardiology continues to be one of the “gold standards” in detecting myocardial ischemia. For example, gated SPECT (single-photon emission computed tomography) continues to play an important role in the diagnosis and risk stratification of patients with CAD; coronary CT is another gold standard for coronary anatomic non-invasive evaluation.

On the other hand, the impact of this chronic disease on the health system plus the economic cost generated during follow-up are high. In the USA, the cost was already high in the past [4], and in the future it will be even greater [5]. Despite this, imaging tests have their advantages [6]. At present, there are different simulated studies on the cost-effectiveness ratio (CER) between stress electrocardiogram and SPECT in the diagnosis and treatment of CAD. However, few non simulated cost-effectiveness studies include large cohorts of patients with a lengthy follow-up in clinical practice to study the CER of myocardial perfusion SPECT and coronary revascularization (CR) in relation to total mortality (TM) and cardiac events (CEs: non-fatal myocardial infarction (MI) and/or cardiac death) [7,8]. Recently, 8496 patients with gated SPECT-MPI were analyzed [8], and it was observed that the combined stress electrocardiogram and SPECT-MPI results allow differentiation between patient groups, where the PCI and CABG are more cost effective in different economic frameworks. The CR is more cost effective in patients with electrocardiographic and scintigraphic ischemia. PCI is more cost effective than CABG [8].

There is a great variety of individual and circumstantial factors that influence the evaluation of the follow-up of patients with scCAD, and we have multiple non-invasive tests (stress echocardiography, radionuclide myocardial perfusion imaging, cardiac magnetic resonance, and coronary computed-tomography angiography) to stratify the risks. Thus, planning for cardiac risk assessment during follow-up has two phases: a cross-sectional phase and a longitudinal phase.

### 2.1. Cross-Sectional Assessment

During the follow-up, a cross-sectional assessment is carried out for patients with scCAD. That is, the patient is evaluated at that moment and according to the type of CAD, risk level, appropriate criteria for the practice of an imaging test, prognostic variables, and clinical situation. Medical decisions are taken in relation to their treatment or whether to continue testing to improve the risk re-stratification. Thus, the cross-sectional assessment has five stages (Figure 1) as described below.

#### 2.1.1. First Stage: Type of Patients

From a clinical point of view, there are different types of patients in relation to their CAD: patients with INOCA (ischemia with non-obstructive coronary arteries), MINOCA (MI with non-obstructive coronary arteries), without MI plus CR, with MI plus RC, and patients with heart failure. The majority of patients referred for assessment for angina do not have obstructed coronary arteries [9]. Nearly 39% of the patients are selected for a coronary angiography because of suspected angina and/or a positive stress test that has non-obstructive CAD; and INOCA is more frequent in women (50–70%) than in men (30–50%) [10]. There are different types of INOCA: microvascular angina, vasospastic angina, both, and non-flow-limiting CAD (diffuse atherosclerosis, <50% stenosis severity by visual assessment) [9].

Patients with MINOCA constitute 6% to 14% of all those with acute MI [11]. The patients with MINOCA are distinct from patients with acute MI with the classic culprit lesion (stenosis >50%) by having lower prevalence of the traditional cardiac risk factors and a lower but clinically significant annual mortality rate [12]. In the VIRGO (Variation in Recovery: Role of Gender on Outcomes of Young AMI Patients) study, young patients with MINOCA were more likely to be women who had a heterogeneous mechanistic profile and had clinical outcomes that were comparable to MI patients with revascularization or plaque ≥50% [12].

In revascularized patients with or without previous MI, a considerable proportion of patients require repeat revascularization procedures during long-term follow-up; and the need to repeat revascularization has a major impact on the patient’s quality of life and is associated with a significant economic burden [13]. The Consensus Document about the management of myocardial revascularization failure from an expert panel of the European Association of Percutaneous Cardiovascular Interventions (EAPCI) provides a broad overview of the clinical management of myocardial revascularization failure with a focus on the three key underlying mechanisms leading to repeat revascularization: (1) failure of percutaneous coronary interventions, (2) failure of coronary artery bypass grafting, and (3) progression of coronary artery disease in previously untreated native coronary segments [13].

CAD is the most common cause of heart failure with reduced ejection fraction and heart failure with preserved ejection fraction [14]. The patients with heart failure with preserved ejection fraction have more cardiac hypertrophy, epicardial CAD, coronary microvascular rarefaction, and myocardial fibrosis. Each of these findings may contribute to the left ventricular diastolic dysfunction and cardiac reserve function impairment characteristic of heart failure with preserved ejection fraction [15]. Thus, all these characteristics in relation to the type of CAD influence the follow-up and management of patients with scCAD.

#### 2.1.2. Second Stage: Clinical Coronary Risk Stratification

In patients with scCAD, we are constantly re-stratifying coronary risk throughout the lives of these patients. This risk assessment is very complex, and multiple aspects must be taken into account. There are a variety of individual factors and different algorithms to assess the risk of these patients over time in relation to cardiac death, non-fatal MI, heart failure, stroke, and coronary revascularization. Generally, the first risk stratification occurs without cardiac imaging or stress testing.

The Framingham Risk Score can identify the risk of cardiovascular events among patients with established CAD [16]. Sara et al. [16] analyzed 25,519 consecutive patients undergoing PCI across a 17-year period and concluded that the Framingham Risk Score discriminates the risk of long-term secondary events, including cardiac death, MI, and revascularization. The European Society of Cardiology—Systematic COronary Risk Evaluation (ESC-SCORE) can also identify the risk of cardiovascular events among patients with established CAD [17]. Biener et al. [18] studied 693 patients with an ESC-SCORE and observed that the prognostic performance for the prediction of the composite end-point all-cause mortality, acute myocardial infarction, stroke, hospital readmissions for acute coronary syndrome, or decompensated heart failure was comparable between the non-secondary prevention group and the secondary prevention group.

Despite this, one of the most complete and complex algorithms that allows the risk re-stratification in patients with CAD in the absence of non-invasive imaging is the ATP III score (Table 1). This program calculates the Framingham 10-year risk percentage as well as providing treatment guidelines based on the latest clinical data [19]. This score should be applied more frequently in clinical practice. The ATP III has four categories of risk: lower risk (<10%): 0–1 risk factor; moderate risk: ≥2 risk factors; moderately high risk (10% to 20%): ≥2 risk factors; high risk (>20%): coronary heart disease or coronary heart disease risk equivalents.

#### 2.1.3. Third Stage: Appropriate Use Criteria in Cardiac Imaging

The appropriate use criteria are a tool that allows us to apply an imaging test with better performance for diagnostic and/or prognostic purposes in different clinical settings. There is currently a guideline for the appropriate use criteria for each imaging technique: echocardiography [20], radionuclide myocardial perfusion imaging [20], cardiac magnetic resonance [7], coronary computed-tomography angiography [7], and invasive coronary angiography [21]. As already known, the rating panel scores each indicate the following: appropriate (median score 7 to 9), may be appropriate (median score 4 to 6), and rarely appropriate (median score 1 to 3) [20]. In addition, testing was found to be appropriate or may be appropriate for patients within 90 days of an abnormal or uncertain prior result [20]. Appropriate use criteria in cardiac imaging should be applied more rigorously in planning the follow-up of patients with CAD because it also has an impact on cost-effectiveness. In patients with suspected stable CAD, the exercise ECG is a cost-effective first-line test for patients with low likelihood. However, with higher likelihood, non-invasive imaging including radionuclide myocardial perfusion imaging is more cost effective because of its greater accuracy [22]. When the prevalence of CAD is <30%, it is more cost effective to exclude strategies involving stress ECG, and MPS-based strategies are cost effective when the risk of CAD is modest [23]. On the other hand, an inappropriate radionuclide myocardial perfusion imaging led to a six-fold increase in the cost of predicting a single event compared with appropriate or uncertain radionuclide myocardial perfusion imaging. Additionally, inappropriate radionuclide myocardial perfusion imaging is nearly 1.5 times more expensive than appropriate or uncertain radionuclide myocardial perfusion imaging for predicting the need for CR within 6 months [24].

In clinical practice, there are patients with whom only an exercise test is sufficient to stratify the risk of their scCAD, and on the other hand, there are patients who, in addition to the stress test, require one or more cardiac imaging tests (echocardiogram, gated SPECT, PET, magnetic resonance, or coronary CT), that is, a multimodality of cardiac imaging. Echocardiography evaluates cardiac structure and function, valve structure and function, synchrony, ischemia, and myocardial viability. On the other hand, magnetic resonance imaging evaluates cardiac muscle and chamber structure and function, tissue characterization, and myocardial ischemia. Furthermore, cardiac CT evaluates coronary circulation, calcium score, plaque characterization, cardiac valve structure, and coronary flow. Finally, nuclear imaging evaluates myocardial ischemia, systolic and diastolic ventricular function, viability, denervation, synchrony, remodeling, myocardial flow reserve, infection, and metabolism [25]. All these techniques can evaluate the same disease from different points of view.

#### 2.1.4. Fourth Stage: Coronary Risk Stratification with Cardiac Imaging

**Figure 3:**
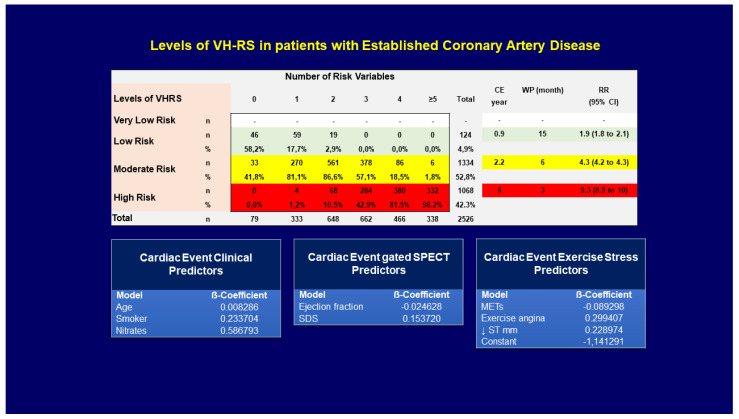
Relationship between VH-RS levels and the number of risk variables. The increase of risk level is associated with an increase in the number of predictor variables. All patients with a high-risk level have one or more risk variables, and the warranty period (WP) of an imaging test for detecting ischemia is very short (approximately 3 months). Very low-risk patients are unlikely to be found among patients with established CAD. CE, cardiac event; SDS, summed difference score; RR, relative risk.

There are multiple algorithms, nomograms, or scores to stratify coronary risk [16,17,18,19,26,27], that have as an objective the calculation of the probability of cardiac death, non-fatal MI, heart failure, stroke, and CR. However, most of them are for patients who do not have previous stable CAD, or analyze the risk without differentiating between patients with established or suspected CAD [26], or are algorithms that do not use an imaging test [16,17,18,19]. Regardless of this, all these studies have their practical application at some point in patient follow-up. The purpose of this review was about planning the assessment of risk in patients with an scEAC, therefore, it requires algorithms, nomograms, or scores validated for patients with previously diagnosed CAD. One of the few extensive studies with 6441 patients recently published to stratify coronary risk is the VH-RS [28]. The Vall d’Hebron Risk Score (VH-RS) method has several particularly useful features: it focuses on patients with different clinical variables, who underwent exercise tests, and myocardial perfusion gated SPECT images, and it assesses an individual’s cardiac risk for non-fatal MI, CR, and/or cardiac death in patients with established CAD or patients with suspected CAD. This new risk score allows the risk to be calculated for individual patients with a mean of a 4-year follow-up. The importance of dividing the population into patients with established CAD or patients with suspected CAD lies in the fact that the patients with established CAD have a significantly higher prevalence of cardiac complications or advanced CAD. The VH-RS allows the stratification of patients into four risk levels: very low risk, low risk, moderate risk, and high risk (Figure 3).

There are two big groups of patients with CAD: patients with ischemia without obstructive CAD and patients with ischemia with obstructive CAD. These different forms of CAD have different mechanisms, and these mechanisms can overlap [12]. Therefore, the analysis of the myocardial flow is very important not only for diagnosis but also for the prognosis value. Traditionally, the coronary flow reserve [29], the fractional flow reserve [30], the hyperemic myocardial velocity resistance [31], and the index of microvascular are evaluated [31] by invasive coronary angiography. However, currently, PET [32] or dynamic gated SPECT [33] allow the assessment of the myocardial flow in a non-invasive way. It is important to consider this non-invasive technology for the follow-up of the patients with microvascular CAD. The same consideration should be given to the fractional flow reserve computation from coronary computed-tomography angiography datasets. Although, fractional flow reserve computation from coronary computed-tomography angiography datasets is probably better for epicardial non-obstructive (stenosis <50%) CAD, and PET or dynamic SPECT are better for patients with microvascular disease [29,30,31,32,33]. More studies are needed to clarify the analysis of coronary flow in these two types of populations.

#### 2.1.5. Fifth Stage: Clinical Decision

The fifth stage corresponds to the final clinical decision whether to continue with the same medical treatment, or to optimize the treatment, or whether the patient requires a new coronary angiography to assess another CR.

### 2.2. Longitudinal Assessment

Usually, during the follow-up of patients with csCAD, repeated medical checks are carried out with different intervals. Thus, the number of times we study a patient throughout the natural history of their disease and the follow-up intervals correspond to the longitudinal analysis of the planning (Figure 2). Predicting the number of times a patient with scCAD will be studied at follow-up is impossible. It depends on each particular case, on multiple clinical factors, and the natural development of the disease. Despite this, the clinical situation of the patients, the associated non-coronary diseases, the controversial warranty periods of a test [34] or of myocardial ischemia [35], and the variables that can predict a second test [36] are tools that help to plan the intervals of follow-up of these patients (Figure 2).

As it is known, at some point in the follow-up of patients with scCAD, it is necessary to re-stratify the risk of CAD using an imaging technique, such as a stress echocardiogram, stress MRI, gated SPECT, or cardiac PET. The reasons for performing these tests can be multiple: reappearance of angina, readmissions, new myocardial infarction, heart failure, or patients who after several years of CR need to be studied again. A very valid tool previously published is the criteria for the appropriate use of an imaging test that allows us to guide ourselves in the application of imaging tests to re-stratify the risk of these patients throughout the follow-up [20]. In clinical practice, each hospital uses different imaging techniques in relation to their availability, experience, cost, and appropriateness for each patient. In this way, a score called the VH-RS was developed, which uses various clinical, stress test, and imaging variables (age, smoking, treatment with nitroglycerin, left ventricular ejection fraction, myocardial ischemic load, angina during exercise, ST elevation during exercise and METs), allowing the grouping of patients with scCAD into low risk, moderate risk, and high risk. Thus, for patients who continue to be at low risk (cardiac event <1%/year), the approximate follow-up interval would be every 15 months. Therefore, this suggests that in low-risk patients with scCAD the follow-up is approximately every year, i.e., routine follow-up in most patients with scCAD. In patients with moderate risk (annual cardiac event 1–2.9%/year), the event can begin to occur 6 months after the study, so it is suggested to carry out a biannual follow-up; and in high-risk patients, the cardiac event can appear after 3 months, so it is suggested to carry out a trimestral follow-up. The moderate- and high-risk groups correspond to patients with a higher risk for non-fatal infarction, cardiac death, and CR due to moderate and severe myocardial ischemia.

Moreover, not all stable, asymptomatic patients with complete revascularization and good left ventricular function will require an imaging test. In these cases, a clinical follow-up without an image, with an electrocardiogram, and a blood test is sufficient. Although we must bear in mind that as time passes, after 4–5 years of successful coronary revascularization, with an asymptomatic patient and good left ventricular function, it would be advisable to study with a non-invasive imaging test [20], since detection of silent ischemia has prognostic value [37,38,39]. If this imaging test is normal, we will continue with clinical controls without imaging tests.

In complex patients, for example in patients with three-vessel CAD treated with multiple PCI, with incomplete revascularization and residual symptoms, it is advisable to assess whether non-revascularized vessels are responsible for angina, myocardial ischemia, or heart failure, using non-invasive imaging techniques (for example fusion imaging), and moreover if these vessels are feasible, to revascularize from an anatomical point of view. In the best of cases, if coronary revascularization is feasible, the clinical picture would improve, and we would carry out annual clinical controls. If despite having achieved a complete RC, the patient remains symptomatic, it would be advisable to conduct a study of coronary flow reserve to assess coronary microcirculation. In the worst case, when a complete CR cannot be achieved due to coronary anatomy or any other cause, we would continue with biannual or triannual clinical controls, as necessary, to try to find the most appropriate medical treatment. Until now we have no tracking guides for these types of patients.

**Figure 4:**
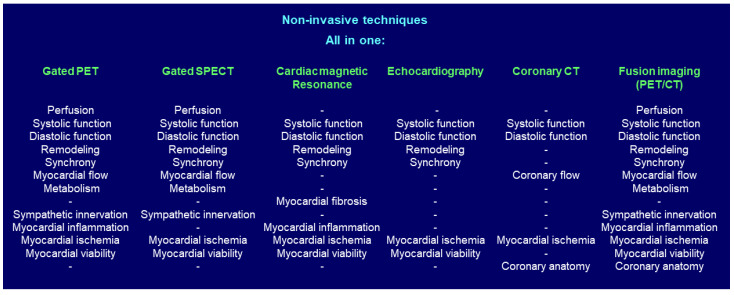
The different non-invasive techniques to evaluate patients with scCAD provide additional information in the same study.

On the other hand, during the follow-up of patients with csCAD, special attention should be paid to eight groups of patients with high morbidity and mortality, with more hospital readmissions and difficult therapeutic management (Table 2). Therefore, the use of non-invasive imaging techniques during follow-up is a useful tool, especially in the case of those that provide multiple data in the same study (gated PET, gated SPECT), as each parameter can provide additional prognostic information (Figure 4).

These different types of CAD have different mechanisms, and these mechanisms can overlap [9]. In patients with INOCA, it is important to have a non-invasive assessment of coronary flow during the follow-up to evaluate the microvascular dysfunction. Radico et al. [37], in a meta-analysis, analyzed 35,039 patients and observed that angina without obstructive CAD has a heterogeneous prognosis. Patients’ quality of life is also worsened by the high incidence of hospitalization, angina recurrence, and repeated coronary angiography. The incidence of all-cause death and non-fatal MI in patients with non-obstructive atherosclerosis is much higher (1.32/100 person-years) than in those with angiographically normal epicardial vessels (0.52/100 person-years).

Recently, Seitz et al. [38] investigated the prognosis of a large cohort of patients with stable angina and unobstructed coronaries undergoing acetylcholine spasm testing, they found that in this long-term follow-up study, the overall prognosis of patients with coronary spasm was favorable. Patients with epicardial spasm have an increased risk for nonfatal MI (HR:14.46) and repeat angiography (HR:1.7), while microvascular spasm was associated with recurrent angina (HR:1.01). Acetylcholine testing may help identify patients at increased risk from adverse cardiac events among this overall low-risk population. However, from the point of view of the clinical follow-up of patients with INOCA, it would be recommended to assess the coronary flow through non-invasive techniques (coronary CT, PET, dynamic gated SPECT), when required. The clinical follow-up of patients with epicardial coronary spasm is performed in relation to the episodes of angina (frequency, duration, appearance circumstance, etc.) and also with ischemic induction tests because most of them have non-significant coronary lesions; and developments in these injuries have to be monitored. Moreover, it is not sufficient to differentiate between coronary spasm angina and progression of a coronary lesion, only with the features of angina.

The prevalence of silent myocardial ischemia (SMI) varies depending on the characteristics of the population (prevalence of CAD, age, gender, and treatment), cardiovascular risk factors, and study design (exercise, pharmacologic, or mixed stress test with or without imaging) [39]. In our series of 1050 patients with SMI, male gender and pharmacologic stress were independent predictors of SMI [39]. Moreover, during the follow-up cardiac events, there were 1.75%/year for SMI and 1.25%/year for symptomatic myocardial ischemia. SMI has an incremental prognosis value over clinical variables and symptomatic ischemia. On the other hand, patients with SMI with only ST-positive results and patients with only SPECT-positive results have similar prognosis, but patients with ST positive plus SPECT positive have worse prognosis [39]. Thus, evaluating patients during the follow-up with an exercise test and gated SPECT, or another imaging study, together should be mandatory.

The extent of CAD may accelerate the onset or progression of heart failure with higher mortality. Although revascularization with thrombolytic agents or percutaneous coronary intervention significantly decrease mortality; left ventricular remodeling may occur despite sustained patency of the infarct-related artery [40]. The assessment of remodeling, metabolism, sympathetic innervation, and ventricular synchrony by nuclear cardiology techniques improves the prognostic assessment of these patients. In addition, the transthoracic echocardiographic could help classify scCAD patients into separate phenogroupings that differentiate cardiopulmonary structural and functional abnormalities and can predict heart failure hospitalization, independent of traditional cardiovascular risk factors [41]. In addition, another complication of CAD is ischemic mitral regurgitation caused by changes in ventricular structure and function with worse prognosis [40].

Diabetes mellitus is a major cardiovascular risk factor with high morbidity and mortality and has a double risk for vascular disease. In diabetic women without known CAD and in diabetic men without known CAD, summed rest score in gated SPECT is the most predictive variable. In diabetic men with known CAD, nitrate treatment is the most predictive variable, and in diabetic women with known CAD, end-diastolic index is the most predictive variable for non-fatal MI, cardiac death, and late CR [42]. There are significantly more major adverse cardiac events (MACEs) in diabetic men than in diabetic women, but the worst prognosis for MACEs occurs in women with known CAD [42].

Patients with chronic kidney disease have high risk of adverse cardiovascular events by multiple mechanisms [43]. In addition, vascular calcification is frequent, and when associated with decreased myocardial flow and coronary flow reserve, the mortality increases too [43]. The presence and severity of myocardial perfusion abnormalities and ischemic burden are associated with a stepwise increase in MACE risk [44]. On the other hand, the coronary artery calcium score, carotid atherosclerotic plaque burden, and myocardial blood flow stress are associated in patients with end-stage renal disease [45]. Atherosclerosis can be detected earlier by myocardial blood flow stress than by coronary flow reserve. Cardiovascular-event-free survival is associated with impaired coronary flow reserve and myocardial blood flow stress [45]. Although recently, in the ISCHEMIA-CKD study, it was observed that among patients with stable CAD, advanced chronic kidney disease, and moderate or severe ischemia, there were no differences between an initial invasive strategy, or an initial conservative strategy, in reducing the risk of death or non-fatal MI [46].

Lower-extremity peripheral artery disease and CAD are both pathologically rooted in atherosclerosis, and their shared clinical features regarding the exposure to cardiovascular risk factors (age, sex, smoking, diabetes mellitus, hypertension, dyslipidemia, and renal failure) have been emphasized [47]. In addition, lower-extremity peripheral artery disease patients are at high risk of future coronary events, as are CAD patients. Data from a Japanese study of 1,121,359 cases; observed that the prevalence of diabetes mellitus and end-stage renal disease is 1.96 and 6.39 times higher in lower-extremity peripheral artery disease patients than in CAD patients [47].

The global population of people ≥80 years of age is currently 137 million and is expected to triple by 2050 [48], and moreover, estimated survival after the age of 80 years is 9.73 years in women and 8.28 years in men [48]. Furthermore, and unfortunately, octogenarians and even-older patients are underrepresented in clinical trials [48]. The coronary vascular age is important, because it has a significant additional value of vascular age over clinical variables and chronological age in predicting the presence of myocardial ischemia [49]. Thus, the communication of a given vascular age would have a superior emotive impact, improving observance of therapies and healthier lifestyles [49]. Additionally, the prevalence of dementia is increasing as the population ages [50]. Dementia is known to share many common risk factors with CAD including age, genetics, smoking, the components of the metabolic syndrome, and inflammation [50].

## 3. Conclusions

Planning the follow-up of patients with scCAD goes beyond the information provided by clinical management guidelines. It requires understanding the importance of a cross-sectional and longitudinal analysis in the clinical history of scCAD, because of its impact on the cost of healthcare in relation to mortality, economic factors, and the burden of medical appointments. Using the data provided in this study facilitates and standardizes the clinical follow-up of patients with scCAD, and following the set marked line makes the work for the clinical cardiologist much easier, by including most clinical possibilities and actions to consider. The follow-up intervals vary according to the clinical situation of each patient and can be highly variable. In addition, the ability to properly study patients with imaging techniques, to stratify at different levels of risk, helps plan the intervals during the follow-up. Given the complexity of coronary artery disease and the diversity of clinical cases, more studies are required in the future, focusing on improving the planning of follow-up for patients with scCAD. The perspective and future direction are related to the valuable utility of integrated imaging techniques in clinical follow-up.

## Figures and Tables

**Table 1:** Variables used in adult treatment panel (ATP III) calculator.

Age
Gender
Smoking status
Systolic blood pressure
Total cholesterol
Triglyceride level
High-density lipoprotein
Low-density lipoprotein
Medication for blood pressure
Are any of the following present? (Yes or No)
1. Clinical coronary heart disease:
Myocardial infarction,
Unstable angina or stable angina,
Coronary artery procedures (angioplasty or bypass surgery),
Evidence of clinically significant myocardial ischemia.
2. Coronary heart disease risk equivalents: includes clinical manifestations of non-coronary forms of atherosclerotic disease:
Peripheral arterial disease,
Abdominal aortic aneurysm,
Carotid artery disease (transient ischemic attacks or stroke of carotid origin or >50% obstruction of a carotid artery),
Diabetes, and
≥2 risk factors with 10-year risk for hard coronary heart disease >20%.
How many of the following risk factors are present if any? (Score: 1,2,3,4,5) (Note: if high-density lipoprotein ≥60, subtract one from the total count).
Cigarette smoking,
Hypertension (BP ≥ 140/90 mmHg or on antihypertensive medication),
Low high-density lipoprotein cholesterol (<40 mg/dL),
Family history of premature coronary heart disease (coronary heart disease in male first-degree relative < 55 years, coronary heart disease in female first-degree relative <65 years),
Age (men ≥45 years; women ≥ 55 years).
Metabolic syndrome. Are at least three of the following present after 3 months of therapeutic lifestyle changes? (Yes: ≥3 criteria met; or No: <3 criteria met).
1. Abdominal obesity (waist circumference)
Women: >88 cm (>35 in).
2. Triglycerides ≥ 150 mg/dL.
3. High-density lipoprotein cholesterol
Women: <50 mg/dL.
4. Blood pressure: ≥130/≥85 mmHg.
5. Fasting glucose: ≥110 mg/dL.

**Table 2:** Prognostic clinical variables corresponding to appropriate use criteria for an imaging test in special groups of patients with chronic stable coronary artery disease.

Microvascular Angina	Vasospastic Angina	Silent Myocardial Ischemia	Heart Failure
Persistent chest pain	Repeated angina episodes	Male	Differentiate cardiopulmonary structural and functional abnormalities
Smoking	Non-fata lmyocardial infarction	Diabetes	
Diabetes	Repeated angiographies	Pharmacologic stress test	
Increased QTc interval		ST segment depression	
**Diabetes**	**Renal Failure**	**Peripheral** **Arterial Disease**	**Advanced Age**
Gender	Age	Diabetes	Elderly patients
Age	Diabetes	End-stage renal disease	Arrhythmias
Insulin treatment	End-stage renal disease	Coronary risk factors	Dementia
Nitrates	Vascular calcification		Coronary risk factors
Hypercholesterolemia	Carotid plaque burden		
Known coronary artery disease			
Peripheral artery disease			
Dipyridamole stress			

## Data Availability

Not applicable.
